# Health-related quality of life as predictor for mortality in patients treated with long-term mechanical ventilation

**DOI:** 10.1186/s12890-018-0768-4

**Published:** 2019-01-11

**Authors:** Heidi Markussen, Sverre Lehmann, Roy M. Nilsen, Gerd K. Natvig

**Affiliations:** 1 The Norwegian National Advisory Unit on Longterm Mechanical Ventilation, Department of Thoracic Medicine, Haukeland University Hospital, Jonas Lies vei 65, N-5021 Bergen, Norway; 2 Department of Global Public Health and Primary Care, University in Bergen, Kalfarveien 31, 5018 Bergen, Norway; 3 Department of Clinical Science, University in Bergen, Bergen, Norway; 4 Faculty of Health and Social Sciences, Western Norway University of Applied Sciences, Inndalsveien 28, 5063 Bergen, Norway

**Keywords:** Long-term mechanical ventilation, Health-related quality of life, Predictors, Mortality, Survival, The severe respiratory insufficiency (SRI) questionnaire

## Abstract

**Background:**

The Severe Respiratory Insufficiency (SRI) questionnaire is a specific measure of health-related quality of life (HRQoL) in patients treated with long-term mechanical ventilation (LTMV). The aim of the present study was to examine whether SRI sum scores and related subscales are associated with mortality in LTMV patients.

**Methods:**

The study included 112 LTMV patients (non-invasive and invasive) from the Norwegian LTMV registry in Western Norway from 2008 with follow-up in August 2014. SRI data were obtained through a postal questionnaire, whereas mortality data were obtained from the Norwegian Cause of Death Registry. The SRI questionnaire contains 49 items and seven subscales added into a summary score (range 0–100) with higher scores indicating a better HRQoL. The association between the SRI score and mortality was estimated as hazard ratios (HRs) with 95% confidence intervals (95% CI) using Cox regression models and HRs were estimated per one unit change in the SRI score.

**Results:**

Of the 112 participating patients in 2008, 52 (46%) had died by August 2014. The mortality rate was the highest in patients with chronic obstructive pulmonary disease (75%), followed by patients with neuromuscular disease (46%), obesity hypoventilation syndrome (31%) and chest wall disease (25%) (*p* < 0.001). Higher SRI sum scores in 2008 were associated with a lower mortality risk after adjustment for age, education, hours a day on LTMV, time since initiation of LTMV, disease category and comorbidity (HR 0.98, 95% CI: 0.96–0.99). In addition, SRI-Physical Functioning (HR 0.98, 95% CI: 0.96–0.99), SRI-Psychological Well-Being (HR 0.98, 95% CI: 0.97–0.99), and SRI-Social Functioning (HR 0.98, 95% CI: 0.97–0.99) remained significant risk factors for mortality after covariate adjustment. In the subgroup analyses of patient with neuromuscular diseases we found significant inverse associations between some of the SRI subscales and mortality.

**Conclusions:**

SRI score is associated with mortality in LTMV-treated patients. We propose the use of SRI in the daily clinic with repeated measurements as part of individual follow-up. Randomized clinical trials with interventions aimed to improve HRQoL in LTMV patients should consider the SRI questionnaire as the standard HRQoL measurement.

**Electronic supplementary material:**

The online version of this article (10.1186/s12890-018-0768-4) contains supplementary material, which is available to authorized users.

## Background

Chronic hypercapnic respiratory failure (CHRF) is a persistent state in which ventilation is insufficient to maintain a normal arterial pressure of carbon dioxide (PaCO_2_) [1, 2]. Long-term mechanical ventilation (LTMV) is a treatment for patients with CHRF due to different aetiologies and includes both non-invasive and invasive mechanical ventilation [2–4]. In adults, CHRF is mainly caused by the following four disease categories: neuromuscular diseases (NMD), chest wall diseases (CWD), obesity hypoventilation syndrome (OHS), and chronic obstructive pulmonary disease (COPD) [2–4]. The number of individuals treated with LTMV is increasing, and the largest growth has been observed in the use of a non-invasive connection to the ventilator through a mask or a mouthpiece [2, 3]. One of the main goals of LTMV is to improve survival [2, 4]. Due to ethical reasons, few randomized controlled trials (RCTs) comparing LTMV versus no LTMV treatment have been carried out in these patients [2, 3]. One of the exceptions is RCTs involving COPD patients with CHRF, where the benefit of LTMV on survival has been and continues to be debated [3, 5, 6]. Two recent RCTs found improved one-year mortality in COPD patients treated with non-invasive LTMV [7, 8]. In NMD patients LTMV has been well-established for several decades [2–4]. One of the few RCTs in this heterogenic category found improved mortality in patients with amyotrophic lateral sclerosis (ALS) [9].

Additionally, several observational studies and uncontrolled trials indicated that LTMV has a positive effect on survival in patients with NMD [10–16], OHS [10, 13, 17–20] and CWD [10–13, 21–23] relative to historical controls.

Self-reported health or health-related quality of life (HRQoL) has been shown to provide prognostic information for different groups [11, 24–27]. The Severe Respiratory Insufficiency (SRI) questionnaire was developed to specifically measure patient-reported HRQoL in patients receiving LTMV [28]. The role of SRI in predicting mortality in patients with CHRF has been examined during two three-year follow-up studies [29, 30]. In the first study, the clinical variables body mass index (BMI), leukocytes, base excess, forced expiratory volume in one second (FEV_1_), and inspiratory vital capacity were included in the multivariate analysis [29]. The SRI score was associated with mortality in all patients except for those with COPD [29]. The second study found significant relationships between the SRI score and three-year mortality in LTMV patients with COPD and pulmonary tuberculosis sequelae after adjustment for BMI, PaCO_2_ and forced vital capacity (FVC), but without subgroup analyses for the different diagnosis group [30]. Other measures of HRQoL, such as St. George’s Respiratory Questionnaire (SGRQ) and the Maugeri Respiratory Failure Questionnaire (MRF-28), were associated with mortality in LTMV-treated COPD patients from 21 study centres during 3 years of follow-up [26]. The SRI score’s ability to predict mortality in patients treated with LTMV has been poorly investigated. Furthermore, findings are inconclusive [29, 30] and the associations have been investigated for only a limited time period (up to three years). Longer follow-up time might capture a more robust association due to higher mortality rates over time.

## Methods

The main objective of the current study was to examine the association between HRQoL measured by the SRI questionnaire and all-cause mortality in LTMV patients over 80 months follow-up.

### Study population

This study drew on resources from the Norwegian Registry for LTMV [31], the Norwegian Patient Registry [32], and the Norwegian Cause of Death Registry [33]. The registry data were linked by the personal identity number provided to all Norwegian citizens. The study was approved by the Regional Committee for Medical and Health Research Ethics number (273.06, 2012/1090–11) and the Norwegian Centre for Research Data (project number 16001). A written consent was a prerequisite from the Regional Committee for Medical and Health Research Ethics and the NPR to allow linking data between the registries. For cohort patients who died, exemption from the consent requirement for register connection to the Norwegian Patient Registry and Cause of Death Register was given.

The Norwegian Registry for LTMV was established in 2002 at Haukeland University Hospital, Bergen. The registry includes all patients in Norway who are treated with LTMV on a daily basis. The registry contains detailed information on patient characteristics, medical diagnosis, LTMV treatment and lung function. The registry has been described in detail previously [34, 35].

During the period of March to June 2008, patients in the Norwegian LTMV registry in Western Norway were invited to participate in the current study. The inclusion criteria were patients treated with non-invasive or invasive LTMV, over 18 years old and mentally able to answer additional study questions. Well-adapted LTMV treatment for at least 3 months was required for all participants. The invitation letter also included the SRI questionnaire, a form with questions on socioeconomic demographic conditions, and questions whether the patient had received help with completing the information.

**Fig. 1:**
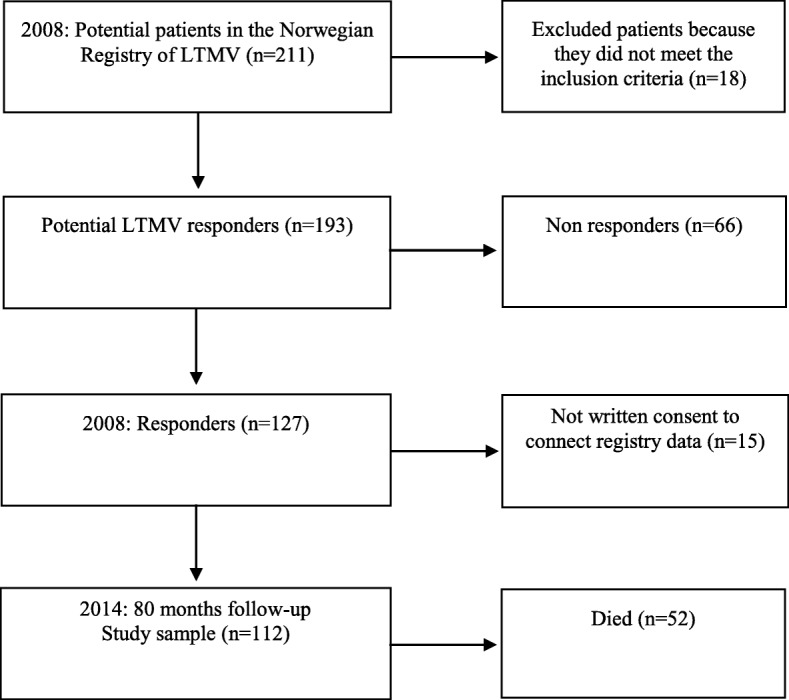
Flow diagram of patients treated with long-term mechanical ventilation in the prospective cohort study from 2008 to 2014. Follow-up time was 80 months

Of 211 potential patients in the LTMV registry, 18 patients did not meet the inclusion criteria (Fig. 1). The remaining 193 eligible patients were invited to participate in the study by postal mail, and 65% (*n* = 127) consented to participate [34]. Of the 127 patients eligible for follow-up, written consent to connect comorbidity data from the NPR were not available from 15 of the patients. The reason for this were dementia or unable to answer the question (*n* = 3), had stopped using LTMV (*n* = 8), unable to make contact (*n* = 1) or did not want to respond (*n* = 3). The disease category for these patients were NMD (*n* = 6), COPD (*n* = 2), OHS (*n* = 6) and CWD (*n* = 1), leaving a final study sample of 112 LTMV patients.

### The severe respiratory insufficiency (SRI) questionnaire

Study participants were asked to complete the SRI questionnaire, which is a multidimensional questionnaire covering physical, psychological and social functioning. It was developed with a comprehensive methodology by physicians specialized in pulmonology and psychologists specialized in HRQoL and by open interviews with patients with CHRF treated with LTMV. The SRI questionnaire contains 49 items, and each item is categorized in one of the following seven subscales: SRI-Respiratory Complaints, SRI-Physical Functioning, SRI-Attendant Symptoms and sleep, SRI-Anxiety, SRI-Social Relationships, SRI-Social Functioning, or SRI-Psychological Well-Being. The subscales were added into a summary scale in which high values (range 0–100) indicate a better HRQoL [28]. The SRI questionnaire demonstrates very good psychometric qualities and has been validated and translated into several languages [2, 34–40]. The SRI and MRF-28 questionnaires were recommended for research on HRQoL in patients treated with LTMV [41]. However, the reliability and validity in were better in the SRI compared to MRF-28 Questionnaire, Clinical COPD Questionnaire and Chronic Respiratory Questionnaire in patients with severe COPD treated with non-invasive LTMV [42]. The responsiveness of the SRI to changes in HRQoL after initiating non-invasive LTMV was superior to the generic questionnaire Short form-36 [43] and the SRI had the best ability to predict mortality compared to other HRQoL questionnaires [30]. The English validation study of the SRI included both non-invasive and invasive (tracheostomy) ventilated LTMV patients [37].

### All-cause mortality

Information on the date of death was obtained from the Norwegian Cause of Death Registry in October 2014. The Cause of Death Registry covers all deaths in Norway and the deaths of Norwegian citizens who die abroad [33]. All deaths (approximately 40,000 each year) are reported by doctors, who are required to complete a standardized death certificate for each death [33].

### Other variables

Based on previous research we also obtained data on educational level [44] and marital status [45]. Educational level was categorized as primary school, high school or college/university, and marital status was classified as married/cohabiting or single/divorced/widowed. Ventilator dependency was reported by the patients in hours a day they used the ventilator, the answer options were categorized as follow; less than 8 h, 8–12, 12–24 h a day.

From the LTMV registry, we collected data on patient age, sex, treatment time since initiation of LTMV, FVC, FEV_1_, PaCO_2_, partial pressure of arterial oxygen (PaO_2_) and main medical diagnosis, which was further categorized into NMD, COPD, OHS, and CWD. Studies have shown that comorbidity is a major prognostic factor in LTMV patients with NMD [13], COPD [46], OHS [18] and CWD [22]. Data concerning comorbidity were not available in the LTMV registry and were therefore collected from the Norwegian Patient Registry (NPR) [33], In this study, comorbidity was assessed similar to another study [47], as the number of somatic ICD-10 diagnosis codes at hospital discharge or an outpatient control for each patient during the recruitment period from March to June 2008.

### Statistical analysis

Patient characteristics were quantified using descriptive statistics. The description was performed according to mean SRI sum score and to mortality status. We used the Kaplan-Meier survivor function with the log-rank test to describe the percentage of survivors according to disease groups (NMD, COPD, OHS and CWD).

The association between SRI and mortality was estimated as hazard ratios (HRs) with 95% confidence intervals (95% CIs) using Cox regression models and HRs were estimated per one unit change in the SRI score. The time in months from study inclusion in 2008 (when baseline SRI was measured) until death was used as a measure of event-free time. All patients were followed up to 80 months until censoring, with August 30, 2014, as the final day of follow-up. We verified that the proportional hazards assumption was fulfilled for SRI, both in overall analyses and in subgroup analyses of NMD, COPD and OHS, by visual inspection of log-log plots. Subgroup analyses of CWD were not performed due to the small sample size.

The HRs with 95% CIs were estimated both by crude and adjusted Cox regression models to control for variables that may potentially confound the true association between SRI and mortality. The adjustment variables included age, education, hours a day on LTMV, treatment time since initiation of LTMV, main disease category and comorbidity. We also evaluated FEV_1_ and FVC as confounding factors in the overall analyses of SRI. To avoid model overfitting in subgroup analysis of disease categories, only the most important covariates were included in the regression models (for NMD: age, hours a day on LTMV, and comorbidity; for COPD: age and comorbidity; for OHS: comorbidity only).

All statistical analyses were carried out using SPSS version 20 (SPSS Inc., Chicago, IL, USA) and Stata SE 14 (StataCorp LP, College Station, TX, USA) for Windows. All statistical tests were two-sided, and *p* values lower than 0.05 were considered to be statistically significant.

## Results

### Background characteristics

The study sample comprised 112 LTMV-treated patients. Of these patients, 48 (43%) were diagnosed with NMD, 24 (21%) with COPD, 32 (29%) with OHS, and 8 (7%) with CWD. At baseline, 103 (92%) patients received non-invasive LTMV, whereas 9 (8%) patients, with NMD, were ventilated invasively via tracheostomy. The mean BMI (*n* = 71) was 27.1 (9.5) in NMD patients, 29.2 (9.1) in COPD patients, 40.6 (7.7) in OHS patients, and 22.5 (5.1) kg/m^2^ in CWD patients.

The baseline mean SRI sum score varied considerably by sex, age group, education level, LTMV hours a day, years on LTMV, disease category and comorbidity (Table 1). The SRI sum score in invasively (*n* = 10) and non-invasively (*n* = 117) ventilated patients was 57.0 (16.2) and 58.0 (18.8), respectively. Assistance to complete the SRI questionnaire was reported by 26% of the study participants.

**Table 1:** Baseline sum score of the Severe Respiratory Insufficiency questionnaire by background characteristics in 112 patients treated with long-term mechanical ventilation between 2008 and 2014

Characteristic	Participants, n^a^ (*n* = 112)	SRI-SS Baseline (2008) mean (SD)
Sex		
Female	48	51.4 (16.7)
Male	57	57.7 (19.5)
Age		
≤ 60	37	59.0 (16.7)
> 60	68	52.5 (19.2)
Education		
Primary school	31	50.0 (20.5)
High school	41	52.2 (16.0)
College/university	33	62.5 (17.6)
Marital status		
Married /cohabiting	60	54.7 (19.1)
Single/divorced/widowed	45	54.9 (18.0)
LTMV h/day		
5–8	45	57.9 (18.3)
8–12	42	55.2 (18.8)
12–24	16	44.9 (17.1)
Years on LTMV		
≤ 4	60	51.9 (18.2)
> 4	45	58.6 (18.4)
Disease		
NMD	43	60.0 (14.8)
COPD	23	41.1 (18.3)
OHS	31	58.0 (18.5)
CWD	8	54.3 (21.1)
Co-morbidity		
No additionally diagnosis	41	60.2 (17.6)
1 additionally diagnosis	28	55.7 (17.9)
≥ 2 additionally diagnosis	36	48.0 (18.3)

Abbreviations: SRI-SS, Severe Respiratory Insufficiency sum score; SD, standard deviation; CI, confidence interval; LTMV, long-term mechanical ventilation; NMD, neuro muscular disease; COPD, chronic obstructive pulmonary disease; OHS, obesity hypoventilation syndrome; CWD, chest wall diseasese

^a^ Numbers do not add to 112 due to missing in the Severe Respiratory Insufficiency questionnaire, as well as missing in education level, marital status and daily hours on LTMV responses

Among the respiratory variables, baseline FEV_1_ and FVC correlated significantly with all SRI subscales except for SRI-Attendant symptoms and sleep scale and SRI-Social Functioning (Additional file 1: Table S1). Baseline PaO_2_ correlated significantly with SRI-Physical Functioning only. All participants were receiving ventilation treatment at study start, and PaCO_2_ levels were therefore normalized at baseline. An inverse correlation between baseline PaCO_2_ and SRI-Social Relationships was present, but no other associations were found for the SRI sum score or for any of the six remaining SRI subscales (Additional file 1: Table S1).

**Fig. 2:**
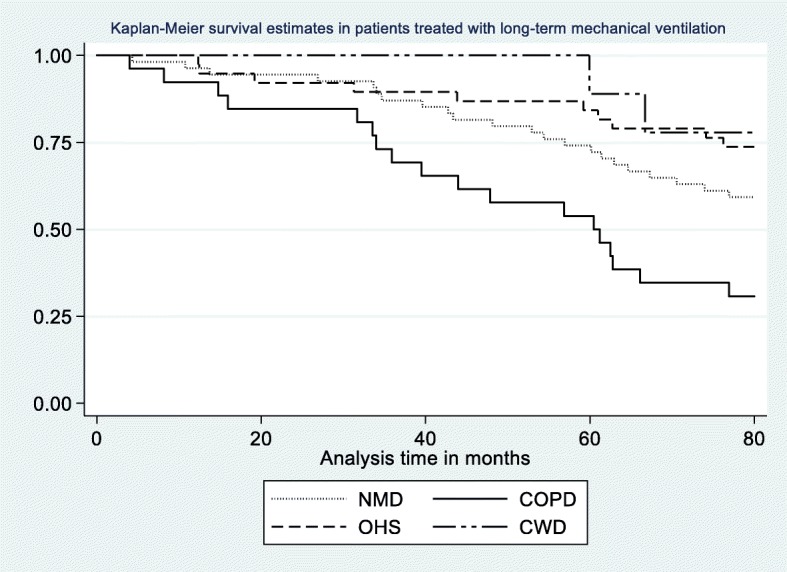
Kaplan-Meier survival estimates in patients treated with long-term mechanical ventilation between 2008 and 2014. Follow-up time was 80 months. Abbreviations: NMD, neuro muscular disease; COPD, chronic obstructive pulmonary disease; OHS, obesity hypoventilation syndrome; CWD, chest wall diseasese

During the 80 months of follow-up, 52 (46%) patients died (Fig. 1). By Kaplan-Meier survival analyses (Fig. 2), we found that patients with COPD had the highest overall mortality rate (75%), followed by patients with NMD (46%), OHS (31%) and CWD (25%) (*p* < 0.001) (Fig. 2). The mortality rates differed between age groups, education levels, LTMV hours a day, years on LTMV, disease categories and burden of comorbidity (Table 2), but not between men and women (*p* = 0.88), and between married /cohabiting and single/divorced/widowed (*p* = 0.91). We found significant differences between survivors and deceased patients in baseline mean FEV_1_ and FVC (both p < 0.001), and a minor difference in PaO_2_ that was not statistically significant (Table 2). There was no significant difference in PaCO_2_ between the survivors and deceased patients (Table 2).

**Table 2:** Background variables at baseline in the survivors and deceased individuals treated with LTMV between 2008 and 2014

Characteristic	All participants, n (*n* = 112)	Survivors, n (%) (*n* = 60)	Deceased, n (%) (*n* = 52)
Sex			
Female	53	28 (53)	25 (47)
Male	59	32 (54)	27 (46)
Age			
≤ 60	38	26 (68)	12 (32)
> 60	74	34 (46)	40 (54)
Education			
Primary school	33	18 (55)	15 (45)
High school	44	23 (52)	21 (48)
College/university	35	19 (54)	16 (46)
Marital status			
Married /cohabiting	64	34 (53)	30 (47)
Single/divorced/widowed	48	26 (54)	22 (46)
LTMV h/day^a^			
5–8	50	33 (66)	17 (34)
8–12	42	23 (55)	19 (45)
12–24	17	2 (12)	15 (88)
Years on LTMV			
≤ 4	65	33 (51)	32 (49)
> 4	47	27 (57)	20 (43)
Disease			
NMD	48	26 (54)	22 (46)
COPD	24	6 (25)	18 (75)
OHS	32	22 (69)	10 (31)
CWD	8	6 (75)	2 (25)
Co-morbidity			
No additionally diagnosis	44	32 (73)	12 (27)
1 additionally diagnosis	29	18 (62)	11 (38)
≥ 2 additionally diagnosis	39	10 (26)	29 (74)
Respiratory^a^			
FVC (litre) (mean, SD)	89	2.64 (1.15)	1.89 (0.93)
FEV1(litre) (mean, SD)	90	1.88 (1.04)	1.16 (0.70)
PaCO_2_ kPa daytime (mean, SD)	84	5.61 (0.76)	6.01 (1.06)
PaO_2_ kPa daytime (mean, SD)	69	10.1 (1.78)	9.14 (1.92)

Abbreviations: LTMV, long-term mechanical ventilation; NMD, neuro muscular disease; COPD, chronic obstructive pulmonary disease; OHS, obesity hypoventilation syndrome; CWD, chest wall disorders; FVC, forced vital capacity; FEV_1,_ forced expiratory volume in one second; PaCO_2_, partial pressure of arterial carbon dioxide; PaO_2_, partial pressure of arterial oxygen

^a^ Numbers do not add to 112 due to missing in daily hours on LTMV and respiratory variables

### SRI sum and subscales in relation to mortality

Crude analyses of baseline SRI showed significantly higher mean values among the survivors compared to the deceased for the SRI sum score and SRI subscales except in the SRI-Attendant symptoms and sleep scale (Table 3).

**Table 3:** Mean baseline scores (both sum score and subscales) of the Severe Respiratory Insufficiency questionnaire in patients treated with long-term mechanical ventilation between 2008 and 2014

SRI	Survivors (*n* = 60)			Deceased (*n* = 52)	*P* value^b^
	n^a^	mean (SD)	n^a^	mean (SD)	
SRI- Sum Score (SS)	58	60.0 (18.3)	47	48.4 (16.8)	0.001
SRI- Physical Functioning (PF)	59	45.9 (23.0)	50	26.9 (21.3)	< 0.001
SRI- Respiratory Complaints (RC)	59	61.0 (22.4)	50	51.1 (22.6)	0.02
SRI- Attendant Symptoms and Sleep (AS)	59	53.3 (20.1)	50	55.8 (20.2)	0.53
SRI- Social Relationships (SR)	59	70.7 (24.4)	49	59.0 (21.8)	0.01
SRI- Anxiety (AX)	59	64.2 (27.5)	49	52.9 (27.2)	0.03
SRI- Psychological Well-Being (WB)	58	66.2 (22.0)	47	52.3 (22.9)	0.002
SRI- Social Functioning (SF)	59	56.5 (24.6)	49	39.1 (18.7)	< 0.001

Abbreviations: SRI, Severe Respiratory Insufficiency; SD, standard deviation

^a^ Numbers do not add to 60 and 52 due to missing in the Severe Respiratory Insufficiency questionnaire

^b^ By two-sample t-test

The association between the SRI sum score and mortality remained significant after adjustment for age, education level, hours a day on LTMV, treatment time since initiation of LTMV, disease category and comorbidity (HR 0.98, 95% CI: 0.96–0.99). In addition, SRI-Physical Functioning (HR 0.98, 95% CI: 0.96–0.99), SRI-Psychological Well-Being (HR 0.98, 95% CI: 0.97–0.99), and SRI-Social Functioning (HR 0.98, 95% CI: 0.97–0.99) remained significant risk factors for mortality after covariate adjustment (Table 4). Additional adjustment for baseline FVC and FEV_1_ did not alter the results much for SRI sum score (adjustment for FVC: HR 0.97, 95% CI: 0.94, 0.99); adjustment for FEV_1_: HR 0.97, 95% CI 0.94, 0.99).

**Table 4:** Hazard ratios for mortality by baseline scores (both sum score and subscales) of the Severe Respiratory Insufficiency questionnaire in all patients (*n* = 112) treated with long-term mechanical ventilation between 2008 and 2014

SRI		Crude			Adjusted^a^		
	N ^b^	HR	95% CI	*P* value	HR	95% CI	*P* value
SRI- Sum Score (SS)	103	0.97	(0.95, 0.98)	0.001	0.98	(0.96, 0.99)	0.04
SRI- Physical Functioning (PF)	107	0.97	(0.95, 0.98)	< 0.001	0.98	(0.96, 0.99)	0.007
SRI- Respiratory Complaints (RC)	107	0.98	(0.97, 0.99)	0.01	0.99	(0.98, 1.01)	0.28
SRI- Attendant Symptoms and Sleep (AS)	107	1.00	(0.99, 1.02)	0.50	0.99	(0.98, 1.01)	0.64
SRI- Social Relationships (SR)	106	0.98	(0.97, 0.99)	0.009	0.99	(0.98, 1.00)	0.14
SRI- Anxiety (AX)	106	0.99	(0.99, 1.00)	0.03	0.99	(0.98, 1.00)	0.25
SRI- Psychological Well-Being (WB)	103	0.98	(0.97, 0.99)	0.001	0.98	(0.97, 0.99)	0.009
SRI- Social Functioning (SF)	106	0.97	(0.96, 0.99)	< 0.001	0.98	(0.97, 0.99)	0.02

Abbreviations: *SRI* Severe Respiratory Insufficiency, *HR* hazard ratio, *CI* confidence interval

^a^ Adjusted for age, education level, daily hours on LTMV, treatment time since initiation of LTMV, disease category and comorbidity

^b^ Numbers do not add to 112 due to missing in the Severe Respiratory Insufficiency questionnaire

Among NMD patients, SRI-Physical Functioning (HR 0.97, 95% CI: 0.94–1.00), SRI-Psychological Well-Being (HR 0.97, 95% CI: 0.95–0.99) and SRI-Social Functioning (HR 0.97, 95% CI: 0.94–0.99) remained significant factors for mortality after adjustment for age, hours a day on LTMV and comorbidity (Table 5).

**Table 5:** Hazard ratios for mortality by baseline scores (both sum score and sub-scales) of the Severe Respiratory Insufficiency questionnaire in neuromuscular patients (*n* = 48) treated with long-term mechanical ventilation between 2008 and 2014

SRI		Crude			Adjusted^a^		
	N^b^	HR	95% CI	*P* value	HR	95% CI	*P* value
SRI- Sum Score (SS)	43	0.97	(0.94, 1.00)	0.09	0.97	(0.93, 1.01)	0.16
SRI- Physical Functioning (PF)	46	0.97	(0.94, 0.99)	0.008	0.97	(0.95, 1.00)	0.05
SRI- Respiratory Complaints (RC)	45	1.00	(0.98, 1.02)	0.88	0.99	(0.97, 1.02)	0.67
SRI- Attendant Symptoms and Sleep (AS)	45	1.01	(0.99, 1.03)	0.20	1.00	(0.98, 1.03)	0.43
SRI- Social Relationships (SR)	45	0.98	(0.96, 1.00)	0.14	0.99	(0.96, 1.01)	0.30
SRI- Anxiety (AX)	45	0.99	(0.98, 1.01)	0.46	0.99	(0.97, 1.01)	0.32
SRI- Psychological Well-Being (WB)	43	0.98	(0.96, 1.00)	0.08	0.97	(0.95, 0.99)	0.03
SRI- Social Functioning (SF)	44	0.96	(0.94, 0.99)	0.002	0.97	(0.94, 0.99)	0.02

Abbreviations: *SRI* Severe Respiratory Insufficiency, *HR* hazard ratio, *CI* confidence interval

^a^ Adjusted for age, daily hours on LTMV and comorbidity

^b^ Numbers do not add to 48 due to missing in the Severe Respiratory Insufficiency questionnaire

In COPD patients, SRI-Attendant Symptoms and Sleep (HR 0.97, 95% CI: 0.94–1.00) and SRI-Psychological Well-Being (HR 0.98, 95% CI: 0.96–1.00) remained associated with mortality after adjustment for age and comorbidity (Table 6). The SRI sum score or subscales were not associated with mortality among patients with OHS (Additional file 1: Table S2).

**Table 6:** Hazard ratios for mortality by baseline scores (both sum score and sub-scales) of the Severe Respiratory Insufficiency questionnaire in chronic obstructive pulmonary disease patients (*n* = 24) treated with long-term mechanical ventilation between 2008 and 2014

SRI		Crude			Adjusted^a^		
	N^b^	HR	95% CI	*P* value	HR	95% CI	*P* value
SRI- Sum Score (SS)	23	0.98	(0.95, 1.01)	0.14	0.97	(0.95, 1.01)	0.19
SRI- Physical Functioning (PF)	23	0.98	(0.95, 1.01)	0.16	0.97	(0.93, 1.01)	0.13
SRI- Respiratory Complaints (RC)	23	0.98	(0.95, 1.01)	0.33	0.98	(0.95, 1.01)	0.26
SRI- Attendant Symptoms and Sleep (AS)	23	0.98	(0.96, 1.01)	0.15	0.97	(0.95, 1.00)	0.10
SRI- Social Relationships (SR)	23	0.99	(0.97, 1.01)	0.08	0.98	(0.99, 1.01)	0.37
SRI- Anxiety (AX)	23	0.99	(0.97, 1.01)	0.32	0.99	(0.97 1.01)	0.34
SRI- Psychological Well-Being (WB)	23	0.98	(0.96, 1.00)	0.07	0.98	(0.96, 1.00)	0.13
SRI- Social Functioning (SF)	23	0.99	(0.97, 1.01)	0.50	0.99	(0.97, 1.01)	0.64

Abbreviations: *SRI* Severe Respiratory Insufficiency, *HR* hazard ratio, *CI* confidence interval

^a^ Adjusted for age and comorbidity

^b^ Numbers do not add to 24 due to missing in the Severe Respiratory Insufficiency questionnaire

## Discussion

We found that HRQoL, as measured by the SRI questionnaire, was inversely associated with mortality in LTMV patients before and after adjustment for covariates. In the total group of LTMV patients, the adjusted analyses showed significant inverse associations between mortality and the SRI sum score and the SRI subscales, ‘physical functioning’, ‘social functioning’ and ‘psychological well-being’. Furthermore, mortality varied considerably between the disease groups during the six-year period. The highest mortality was among COPD patients with established CHRF receiving LTMV. The majority of mortality in COPD is related to cardiac disease and the requirement of LTMV in COPD might be understood as a marker of overall frailty and multi-system disease severity. The lowest mortality was in the CWD group, reflecting the non-progressive nature of the disease in these patients.

As shown in previous studies [3, 10–13, 23], mortality in patients treated with LTMV is associated with underlying disease categories. Previous studies have shown large variations in the attending patient categories, severity of disease, and follow-up times. Thus, a direct comparison of mortality between studies on patients with LTMV is challenging and might lead to an oversimplification.

### HRQoL as a prognostic factor

The association between poor HRQoL and increased mortality in the total group of LTMV patients is consistent with the main findings of other similar studies on LTMV patients [30, 31]. In line with Budweiser (2007a), crude analyses of SRI were significantly associated with mortality in all SRI subscales, with the exception of the ‘attendant symptoms and sleep’ scale.

The adjusted analyses among NMD patients showed that SRI ‘physical functioning’, ‘psychological well-being’ and ‘social functioning’ continued to be significant factors for mortality, which was consistent with the study by Budweiser [30], but with different adjustment variables than those in our study. We also found associations between SRI and mortality among COPD patients in the adjusted analyses in the ‘attendant symptoms and sleep’ and ‘psychological well-being’ SRI subscales.

The initial choice of the adjustment variables in the present study was based on previous work that evaluated age [10, 15, 26, 45], sex [18, 48], education level [44], marital status [45], disease categories (NMD, COPD, OHS and CWD) [2, 3, 10–13, 23] and comorbidity [13, 18, 22, 46]. The variables ventilator dependency and time since LTMV was initiated were chosen a priori. Marital status was not associated with neither mortality nor the SRI sum score and was therefore excluded as adjustment variable. There were no sex differences between survivors and those who deceased, thus sex also was excluded as an adjustment variable.

However, we have considered the possibility that HRQoL could be influenced by other confounding covariates that might also pose a risk of death, such as PaCO_2_. Reduced PaCO_2_ levels have been related to lower one-year mortality and improved SRI scores in COPD patients treated with LTMV [7, 8]. On the other hand, exploratory analyses did not identify any significant correlations between changes in hypercapnia status or baseline hypercapnia status and mortality in this group [5]. However, in the present study, PaCO_2_ values were normalized at baseline as a result of ongoing LTMV and were therefore not included in the analyses. The results from studies on lung function and survival in LTMV patients are not conclusive. Some studies [19, 23, 30] reported associations between low FEV_1_ and FVC and mortality, whereas another study [11] found no differences in baseline lung function between the survivors and deceased patients. When FVC and FEV_1_ were added to the Cox regression analysis in the current study, the result was altered only slightly; however, this result might also be influenced by missing lung function data (FVC baseline numbers did not sum to 112 due to 23 missing data points, FEV_1_ baseline numbers did not sum to 112 due to 22 missing data points), some of the missing data might be explained due to patients having difficulties performing the spirometry test.

We also considered to include ventilation mode as a covariate as longer survival were reported in patients with DMD using non-invasive LTMV compared to those receiving LTMV via a tracheostomy [14, 49]. However, another study concluded that the risk of death was not associated with use of invasive versus non-invasive LTMV in patients with DMD [16], No significant difference in one year mortality was found between patients receiving LTMV via a tracheostomy and those weaned after discharged from the Intensive Care Unit (ICU) and no significant difference in HRQoL measured by SRI at discharge from ICU were found between the two groups [50]. However, HRQoL tended to be lower, in the SRI ‘physical functioning’, while scores for ‘anxieties’ tended to be better in patients receiving LTMV via tracheostomy compared to those treated with non-invasive LTMV [51].

Although the analyses in the present study were adjusted for education level, other economic confounding variables, such as income, might also have an impact on HRQoL and mortality. On the other hand, Norwegian society and health care services probably represent one of the most equitable systems worldwide, where all citizens have equal access to health care services. Nevertheless, the number of covariates that could be included in the analyses in this study was limited by the sample size at baseline, and we can never exhaustively cover all variables of minor importance among LTMV patients.

### Why and how SRI predicts mortality

Previous studies using patient-reported measures other than SRI have also reported an association between self-reported health and mortality in patients treated with LTMV [11, 26]. However, these studies did not adjust for the same covariates as the present study, and they lacked important variables, such as comorbidity and education level. There is a large body of evidence on the association between self-reported health measures and mortality in other settings and disorders, such as in communities [24], in patients with cancer [25] and idiopathic pulmonary fibrosis [27]. Explanations of these consistent findings are complex and imply that survey respondents’ perceptions of health status are holistic; they include information on medical status but that information might be evaluated differently by men and women in different social positions, with different reference groups providing different social comparisons [24]. Further, the accuracy of self-reported health as a predictor of mortality depends on the comprehensiveness and accuracy of the information that the person incorporates into the self-rating [52]. This hypothesis corresponds with SRI as a multidimensional comprehensive questionnaire that captures the symptoms of CHRF and covers essential aspects of LTMV patients’ daily life [28].

### Clinical implication of the associations between SRI and mortality

Individuals suffering from CHRF treated with LTMV often have an incurable disease [2–4]. Health care professionals and relatives tend to behave differently depending on whether the disease is perceived as a chronic or terminal condition. However, the distinction between the patient’s condition as chronic or terminal might become vague and can sometimes be ambiguous and difficult to interpret [53]. Prognostic information from the SRI questionnaire might provide valuable knowledge on how to cope with these situations, improving treatment plans and communication between involved professionals, family members, and the LTMV patient. Our study demonstrates that the risk of death decreases by each unit increase in the SRI score. This result suggests that LTMV patients with low SRI should be identified, initiating thorough considerations on how to improve HRQoL. However, whether the relationship between mortality and quality of life is causal and changes in HRQoL status in some way influences mortality cannot be confirmed in this study design.

The minimal clinically important difference of the SRI questionnaire has not been defined [41]. However, the great numerical difference in SRI score at baseline between the surviving LTMV patients and those who died during the follow-up, support the clinical relevance of the study.

### Strengths and limitations

As far as we are aware, this study is among the very first to examine SRI scores as a predictor for mortality in LTMV patients with a follow-up time as long as 80 months. The strengths of the study include the use of standardized data collection [32–34], including relevant confounders, such as comorbidity, which is often lacking in study of this type, and the prospective study design. Another strength is the use of the specific and validated SRI questionnaire, which can capture HRQoL related to symptoms and the experience of having CHRF and LTMV [2, 28–30, 34–43].

The study has some limitations. First, its small sample size may decrease the statistical power to detect clinically relevant associations in multivariate Cox analyses. Second, comorbidity modeled simply as the number of somatic diagnoses. Charlson Comorbidity Index [54] is a common index to measure comorbidity using ICD-10 codes. However, as complete ICD-10 codes were not available in our data, we chose to measure comorbidity as the number of somatic diagnoses. Thirdly, some of the LTMV patients answered that they received help to complete the questionnaire, which might introduce some information bias in SRI scores. However, it is of great importance to include the SRI scores from patients who needed help to fill out the questionnaire.

In addition, because of the observational study design, we cannot exclude the possibility of residual or unknown confounding. Whether HRQoL score reflects a perception by the LTMV patient of progression of her or his condition or whether change in HRQoL status in some way also influences the course of the condition is an interesting question. However, the research design cannot confirm causality between improvement in HRQoL and survival in this study. To address this question a randomized interventional study aiming to improve HRQoL with a control group receiving standard treatment would be more suitable.

## Conclusion

This study suggests that SRI is an important factor in prognostic mortality models in LTMV-treated patients. The design and data do not allow us to imply any causal relationships between a change in HRQoL and a change in mortality. We propose an active use of the SRI questionnaire in the daily clinic with repeated measurements as part of individual follow-up. Future studies on this topic should be larger and preferably organized as multicentre long-term RCTs, including specific interventions aimed at improving HRQoL in LTMV patients, compared to standard care. Even if there is no comparison in this paper made between SRI and other quality of life measures, we suggest SRI to be used as the quality of life measure in the studies to come.

## Additional file

Additional file 1: **Table S1.** Correlation between baseline scores (both sum score and subscales) of the Severe Respiratory Insufficiency questionnaire and respiratory variables in 112 patients treated with long-term mechanical ventilation between 2008 and 2014. **Table S2.** Hazard ratios for mortality by baseline scores (both sum score and subscales) of the Severe Respiratory Insufficiency questionnaire in obesity hypoventilation syndrome patients (*n* = 32) treated with long-term mechanical ventilation between 2008 and 2014. (DOCX 68 kb)

## Abbreviations

BMI: Body mass index
CHRF: Chronic hypercapnic respiratory failure
CI: Confidence interval
COPD: Chronic obstructive pulmonary disease
CWD: Chest wall disease
FEV_1_: Forced expiratory volume in one second of expiration
FVC: Forced vital capacity
HR: Hazard ratio
HRQoL: Health-related quality of life
LTMV: Long-term mechanical ventilation
NMD: Neuromuscular disease
OHS: Obesity hypoventilation syndrome
PaCO_2_: Arterial partial pressure of carbon dioxide
SD: Standard deviation
SRI questionnaire: Severe Respiratory Insufficiency questionnaire
