# Supplementary material for: Health-related quality of life as predictor for mortality in patients treated with long-term mechanical ventilation
Source: BMC Pulm Med. 2019 Jan 11;19:13. doi: 10.1186/s12890-018-0768-4 (PMC6330471; doi:10.1186/s12890-018-0768-4)
Supplement: Supplementary file 1 — Table S1. Correlation between baseline scores (both sum score and subscales) of the Severe Respiratory Insufficiency questionnaire and respiratory variables in 112 patients treated with long-term mechanical ventilation between 2008 and 2014. Table S2. Hazard ratios for mortality by baseline scores (both sum score and subscales) of the Severe Respiratory Insufficiency questionnaire in obesity hypoventilation syndrome patients (n = 32) treated with long-term mechanical ventilation between 2008 and 2014. (DOCX 68 kb) [file 12890_2018_768_MOESM1_ESM.docx]

Additional file 1: **Table S1**.Correlation between baseline scores (both sum score and subscales) of the Severe Respiratory Insufficiency questionnaire and respiratory variables in 112 patients treated with long-term mechanical ventilation between 2008 and 2014

|  | N^a^ | Correlation coefficient  FVC*^a^* | P  value |  | N ^a^ | Correlation coefficient  FEV_1_*^a^* | P  value | N ^a^ | Correlation coefficient  PaO_2_*^a^* | P  value | |  | N ^a^ | Correlation coefficient  PaCO_2_*^a^* | P  value |
| --- | --- | --- | --- | --- | --- | --- | --- | --- | --- | --- | --- | --- | --- | --- | --- |
| SRI-SS | 83 | 0.29 | 0.008 |  | 84 | 0.24 | 0.03 | 65 | 0.22 | | 0.07 |  | 78 | -0.17 | 0.13 |
| SRI-PF | 86 | 0.28 | 0.009 |  | 87 | 0.27 | 0.01 | 68 | 0.26 | | 0.03 |  | 81 | -0.18 | 0.10 |
| SRI-RC | 86 | 0.32 | 0.003 |  | 87 | 0.36 | 0.007 | 68 | 0.16 | | 0.20 |  | 81 | -0.11 | 0. 33 |
| SRI-AS | 86 | -0.02 | 0.85 |  | 87 | -0.07 | 0.53 | 68 | -0.16 | | 0.17 |  | 81 | 0.04 | 0.73 |
| SRI-SR | 86 | 0.27 | 0.01 |  | 86 | 0.16 | 0.15 | 67 | 0.20 | | 0.11 |  | 80 | -0.23 | 0.04 |
| SRI-AX | 85 | 0.30 | 0.006 |  | 86 | 0.26 | 0.01 | 67 | 0.16 | | 0.20 |  | 80 | -0.17 | 0.14 |
| SRI-WB | 83 | 0.21 | 0.06 |  | 84 | 0.13 | 0.25 | 65 | 0.17 | | 0.19 |  | 78 | -0.14 | 0.23 |
| SRI-SF | 86 | 0.18 | 0.10 |  | 87 | 0.17 | 0.11 | 68 | 0.20 | | 0.23 |  | 81 | -0.15 | 0.18 |

Abbreviations: SRI, Severe Respiratory Insufficiency; SS, sum score; PF, Physical Functioning; RC, Respiratory Complaints; AS, Attendant Symptoms and Sleep; SR, Social Relationships; AX, Anxiety; WB, Psychological Well-Being; SF, Social Functioning; FVC, forced vital capacity; FEV1, forced expiratory volume in one second of expiration; PaO2, partial pressure of oxygen; PaCO2, partial pressure of carbon dioxide.

^a^ Numbers do not add to 112 due to missing SRI and respiratory variables.

*^b^* By Spearman correlation.

Additional file 1: **Table S2**. Hazard ratios for mortality by baseline scores (both sum score and subscales) of the Severe Respiratory Insufficiency questionnaire in obesity hypoventilation syndrome patients (n = 32) treated with long-term mechanical ventilation between 2008 and 2014

| SRI |  |  | Crude | | |  | Adjusted^a^ | | |
| --- | --- | --- | --- | --- | --- | --- | --- | --- | --- |
|  |  | N^b^ | HR | 95% CI | P value |  | HR | 95% CI | P value |
| SRI- Sum Score (SS) |  | 31 | 0.98 | (0.95, 1.02) | 0.32 |  | 1.00 | (0.96, 1.03) | 0.84 |
| SRI- Physical Functioning (PF) |  | 32 | 0.97 | (0.94, 1.00) | 0.07 |  | 0.98 | 0.95, 1.01) | 0.17 |
| SRI- Respiratory Complaints (RC) |  | 32 | 0.98 | (0.95, 1.01) | 0.26 |  | 0.99 | (0.96, 1.02) | 0.61 |
| SRI- Attendant Symptoms and Sleep (AS) |  | 32 | 1.00 | (0.97, 1.04) | 0.74 |  | 1.01 | (0.97, 1.04) | 0.68 |
| SRI- Social Relationships (SR) |  | 31 | 0.99 | (0.96, 1.02) | 0.39 |  | 0.95 | (0.87, 1.03) | 0.99 |
| SRI- Anxiety (AX) |  | 31 | 1.00 | (0.97, 1.02) | 0.83 |  | 1.00 | (0.98 1.03) | 0.46 |
| SRI- Psychological Well-Being (WB) |  | 31 | 0.99 | (0.97, 1.02) | 0.62 |  | 0.99 | (0.97, 1.03) | 0.97 |
| SRI- Social Functioning (SF) |  | 32 | 0.98 | (0.94, 1.00) | 0.15 |  | 0.98 | (0.94, 1.02) | 0.38 |

Abbreviations: SRI, Severe Respiratory Insufficiency; HR, hazard ratio; CI, confidence interval.

^a^ Adjusted for comorbidity.

^b^ Numbers do not add to 32 due to missing in the Severe Respiratory Insufficiency questionnaire.
